# Preparation strategies of mussel-inspired chitosan-based biomaterials for hemostasis

**DOI:** 10.3389/fphar.2024.1439036

**Published:** 2024-08-15

**Authors:** Guihua Cui, Xiaoyu Guo, Li Deng

**Affiliations:** ^1^ Department of Chemistry, Jilin Medical University, Jilin, China; ^2^ Jilin Vocational College of Industry and Technology, Jilin, China; ^3^ Department of Extracorporeal Life Support, The People’s Hospital of Gaozhou, Gaozhou, China

**Keywords:** preparation methodology, mussel-inspired strategy, chitosan-based biomaterials, hemostasis, wound healing

## Abstract

Chitosan (CS) has been extensively studied in wound care for its intrinsic hemostatic and antibacterial properties. However, CS has limiting hemostasis applications on account of its drawbacks such as poor adhesion in humid environments and water solubility at neutral pH. CS-based biomaterials, inspired by mussel-adhesive proteins, serve as a suggested platform by biomedical science. The reports show that the mussel-inspired CS-based hemostatic structure has negligible toxicity and excellent adhesiveness. Biomedicine has witnessed significant progress in the development of these hemostatic materials. This review summarizes the methods for the modification of CS by mussel-inspired chemistry. Moreover, the general method for preparation of mussel-inspired CS-based biomaterials is briefly discussed in this review. This work is expected to give a better understanding of opportunities and challenges of the mussel-inspired strategy for the functionalization of CS-based biomaterials in hemostasis and wound healing. This review is hoped to provide an important perspective on the preparation of mussel-inspired CS-based hemostatic materials.

## 1 Introduction

Chitosan (CS) has been extensively studied in effective bleeding control and wound protection from infection due to its intrinsic hemostatic and antibacterial properties. This is because CS is a natural cationic polysaccharide with abundant amino groups. The surface of erythrocytes and platelets in blood is negatively charged, so CS can induce the aggregation of erythrocytes and platelets, eliminating the electrostatic repulsion between them (Rao and Sharma, 1997; He et al., 2013; Lee et al., 2013). The hemostatic mechanism of CS materials is different from that of the conventional coagulation pathway and does not depend on the patient’s own coagulation pathway and function, which makes it a good material for coagulation and hemostasis. The approved commercially available dressing or gauze of CS by the US Food and Drug Administration (FDA) was Celox, Trauma-Stat, HemCon, PerClot, ChitoGauze, Celox Gauze etc. (Hickman et al., 2018). These products utilize the electric charge of carboxylate and amine groups to bind with tissues and cells (Nainggolan et al., 2018; Simpson et al., 2022). However, the presently available marketed CS hemostatic materials have limitations such as high cost, poor mechanical properties, and water solubility at neutral pH; application of pressure to stanch blood flow due to low adhesion; and tendency to dissolve/deteriorate in the presence of blood (Singha et al., 2020). In addition, these materials cause the injury to rebleed once removed from the site of application. Accordingly, it should be necessary to exploit a new strategy for the improved CS-based hemostatic compositions using functionalization of CS like quaternization and phosphorylation and use of biocompatible cross-linkers for maintaining the structural integrity and enhancing tissue adhesion to achieve rapid hemostasis.

Catechol-functionalized materials are of particular interest because, in nature, mussels secrete (3,4-dihydroxy-L-phenylalanine, DOPA) moiety-rich adhesive proteins, and these proteins exhibit strong water-resistant properties. Inspired by mussels, Lee et al., discovered that the self-polymerization of dopamine (DA) can form surface-adhering polydopamine (PDA) films on the surface of a variety of inorganic and organic materials in 2007 (Lee et al., 2007). After that, mussel-inspired modification strategies have not only been limited to introduce excellent self-adhesiveness as coating materials but have rapidly incorporated into a wide range of applications across the biomedical field. The mussel-inspired CS modification strategy can endow CS-based biomaterials with outstanding adhesive performance, antioxidant property, antibacterial property, coating capacity, high reactivity, chelation and coordination ability, bioactivity and biocompatibility, and also affords a far-reaching platform for the fabrication of various hybrid materials with specific functions. It greatly expands the application of CS-based biomaterials in the field of biomedicine (Li et al., 2021). There are many forms of mussel-inspired CS-based biomaterials, such as dressing, hydrogels, sponge, injectable gels, spray, and powder. In recent years, the mussel-inspired strategy for CS has received extensive attention and mussel-inspired chemistry has created a library of CS-based biomaterials. In this work, we will focus on summarizing the methodology of mussel-inspired modification strategies for CS.

## 2 Mussel-inspired modification strategies for CS

The multiple catechol-mediated interactions provide a significant platform to fabricate versatile mussel-inspired CS-based biomaterials, including catechol-functionalized (such as dopamine (DA), DOPA, norepinephrine, and their derivatives) biomaterials and gallol-functionalized (mainly natural plant polyphenols such as gallic acid and tannic acid (TA)) materials (Zhang RL. et al., 2019; Sanandiya et al., 2019; Shou et al., 2020; Li Y. et al., 2022; Sun CY. et al., 2022; Zhao et al., 2022; Fang et al., 2023). In addition to exploring bionic adhesives, mussel-inspired chemistry also paves the way for the development of new multi-purpose platforms, which are stimulus-responsive materials (Huang et al., 2018; Xu et al., 2019; Li et al., 2021). By making a general survey on the preparation of mussel-inspired CS-based hemostatic materials, it is observed that there are two main strategies for CS modification by mussel-inspired chemistry. One is the PDA or other (which is formed by self-polymerization of dopamine and its derivatives) modified CS; the other is the covalent bonding of catechins or polyphenols and their derivatives onto the CS backbone.

### 2.1 PDA or other products of self-polymerization modified CS

Inspired by the composition of adhesion proteins in mussels, it was found that self-polymerization of dopamine could be used to create new coating materials (Lee et al., 2007). PDA coating technology has attracted more and more attention because of its simplicity and versatility. The initial oxidation can turn the catechol groups of dopamine into quinones. The resulting DA-quinone can undergo intramolecular cyclization and reversible oxidation and then intramolecular rearrangement. The proposed PDA structures were formed by charge transfer, hydrogen bonding, and π–π interactions (Dreyer et al., 2013; Faure et al., 2013; Liu et al., 2014; Patil et al., 2018). It was found that dopamine can undergo oxidative self-polymerization under acidic conditions, and its mechanism is similar to that under alkaline conditions. Self-polymerization of dopamine is particularly suitable for functionalized CS because the PDA coating can further react with amines and sulfates through the Michael addition reaction. Moreover, all the surfaces of hemostatic materials can be unified by a convenient one-step process.

Here are a few typical examples for PDA or other products of self-polymerization modified CS. PDA can exist in the form of nanoparticles (NPs) or thin films or other forms (Lee et al., 2007), and there are two main categories to prepare PDA-NPs: one is to prepare individual PDA NPs and then modify CS. Sun et al., fabricated CS-based alternative absorbable hemostatic sponges that were synthesized by one-step mixing of oxidized dextran (OD), carboxymethyl CS (CCS), and PDA-NPs via the lyophilization method (Sun W. et al., 2022). The results indicated that the interfacial interactions between the functional groups on the OD/CC matrix and the catechol groups on PDA-NPs, which might enhance the pore formation capability of the sponges during the gelation process. The CS-based hemostatic sponges not only accelerated the hemostatic process and prevent bacterial infection but also promoted the healing process, as shown in Supplementary Figure S1. The OD and CS composites were used as scaffolds for multifunctional wound dressing platforms, and their three-dimensional spongy structure made them have good blood and tissue exudate absorption activity and compression elasticity. PDA-NPs serve as a photothermal agent for antimicrobial therapy and an active site for thrombin fixation. Similar to this, Zhang et al. prepared PDA by the DA self-polymerization in a nearly neutral solution at different concentrations and then covalently bound to hydroxybutyl CS to form temperature-responsive CS-based hydrogels (Zhang X. et al., 2019). Gao and Tao also did a similar study (Gao et al., 2019; Tao et al., 2021); the individual PDA-NPs were obtained with a one-pot synthesis and that the aromatic rings on the surface of PDA NPs can adsorb a variety of drugs (ciprofloxacin or curcumin etc.) via π–π stacking and/or hydrogen bonding interactions to form drug-loaded mesoporous polydopamine NPs. Interestingly, the CS-based biomaterials obtained in these two groups were both stimulus-responsive. Near-infrared (NIR) irradiation could activate the photothermal PDA NPs to generate local hyperthermia for antibiosis. The other includes composite NPs containing PDA, and the complexes used dopamine with other substances to form polymers or NPs and then modification. From the literature, DA was directly dissolved in an OD solution to form a PDA@OD complex by self-polymerized PDA interacted with the OD through the Schiff base and non-covalent bonds (Yin et al., 2022), and then, the PDA@OD complex was dispersed in solution and mixed with CS@BSA@DP-NPs (CS-coated bovine serum albumin (BSA)-NPs and loaded dracolin perchloric (DP) acid salt) to obtain CS-based composite nanohydrogels with excellent hemostatic properties. A similar study was conducted by Guo et al., (Qiao et al., 2023). Although the water solubility of poly (thiophene-3-acetic acid) (PTAA) is insufficient, its antibacterial wound dressings are promising. PDA was selected to cover the surface of PTAA to form PDA-coated PTAA (PTAA@PDA), and it can enhance the hydrophilicity of PTAA. PTAA@PDA photothermal properties were also further improved due to the addition of PDA, and it modified carboxymethyl CS in the presence of Fe^3+^ to obtain a novel hemostatic hydrogel for wound healing. It is also very commonly for PDA to overlay metal ions to form NPs, for instance, PDA coating-reduced AgNPs to form PDA@AgNPs@bilayer hydrogels containing CS for photothermal therapy (PTT), and the hydrogel presents adhesiveness due to the catechol group on a PDA molecule. The skin test results demonstrated that the bilayer hydrogel could accelerate infected skin generation by facilitating collagen deposition (Li Y. et al., 2022). In addition, PTAA can also impart electrical conductivity to hydrogel dressings because of electroconductibility. The introduction of PDA into the hydrogel can enable it with both high-efficiency antibacterial properties and conductivity. In addition, PDA-decorated carbon nanotubes (CNTs-PDA) were prepared with excellent dispersion, biocompatibility, and antioxidant properties and cross-linked with boric acid-modified CS to CS-CNT-based conductive wound dressings. The hydrogels could effectively reduce the expression level of wound inflammatory factors; accelerate collagen deposition, epithelial tissue, and vascular regeneration; and thus promote wound healing (Deng et al., 2022). CS/DA/diatom–biosilica composite beads (CDDs) were prepared by the alkalization precipitation method using CS-dopamine mixed solution and diatom–biosilica (DB). It was found that dopamine was oxidized to polydopamine by analyzing the infrared spectra of the beads and formed CDDs together with CS and DB. The polydopamine complex could be oxidized and rearranged to form 5, 6-dihydroxyindoles under the alkaline condition, and the 5, 6-dihydroxyindoles formed dehydrogenized indole carboxylate by intramolecular cyclization or Michael reaction and finally crosslinks to form PDA (Wang et al., 2018). The CDDs exhibited good biocompatibility and hemostatic activity, and its diameter was approximately 1.5 mm; it could also avoid the risk of blockage of capillaries. However, CS blocked the pore structure of DB during the preparation process, and this reduced the porosity of CDDs and weakened the interface interaction between CDDs and blood. Therefore, they improved the experiment by using TBA (tert-butyl alcohol) to replace the water in wet CDDs to obtain CDD-TBA. The reason is that TBA has a high freezing point and which could be completely miscible with water and recover the porosity of DB in the CDD-TBA matrix. More importantly, the surface tension of TBA was lower than that of water, which could reduce the capillary force in the materials to avoid network damage and maintain the 3D network structure of CS-based biomaterials to achieve the desired hemostatic effect (Li et al., 2020).

### 2.2 Grafting catechins or polyphenols and their derivatives to CS backbone

Catechins or polyphenols and their derivatives could be grafted to the main chains of CS through covalent bonds. CS is rich in reactive functional moieties including -NH_2_ and -OH. Furthermore, additional active sites (such as -CHO, -COOH, and -SH) can also be introduced into CS via appropriate chemical modification methods. These functional groups of CS can easily react with catechins or polyphenols and their derivatives through formation amide, imine, ester, and multifarious linkers. The catechol- or gallol-functionalized CS can be endowed with multifunctional properties, such as bio-adhesive/wet adhesion, antimicrobial, biocompatible, anticoagulant, injectable, degradable, antioxidative, angiogenic, and anti-inflammatory properties, which greatly expand their biomedical applications including wound healing, hemostatic, and tissue regeneration (Ong et al., 2008; Qu et al., 2018; Han et al., 2020; Li et al., 2021).

There are so many pathways to form mussel-inspired CS-based biomaterials. CS and its derivatives were grafted with dopamine, such as quaternized CS was grafted with methacrylate, and then, CS-based products were prepared using methacrylate anhydride (MA) as medium (Han et al., 2020; He et al., 2020; Liu et al., 2022; Yang et al., 2022). Inspired by the strong adhesive mechanism of mussels, gallic acid was conjugated to chitosan backbone to obtain a tunicate-inspired hydrogel through the chemical modification of the primary amino groups of CS, as shown in Supplementary Figure S2 (Sanandiya et al., 2019). The adhesion of the tunicate-inspired hydrogel exhibited two-fold greater adhesion ability in the wet condition than did fibrin glue, a commercially available surgical glue. The hemostatic function vis-à-vis the wet adhesiveness of the synthesized chitosan-based material may be useful for facilitating the shortcomings of the restorative tissue medicine. Based on the route of methacrylate (MA)-modified CS, Dai et al. compounded methacrylate anhydride dopamine (DAMA) and Zn-doped whitlockite NPs (Zn-nWH) into methacrylate anhydride-quaternized CS (QCSMA) to obtain a multifunctional hydrogel dressing with hemostasis, disinfection, and wound healing promotion (Yang et al., 2022). The adhesion strength of hydrogel dressing was 0.031 MPa and hemostatic efficiency (129 ± 22s, 27 ± 5 mg) in organism was much higher than that of CS. The other dopamine modified CS hydrogel was fabricated by cross-linking with citric acid (CS-CA-DA) (Liu et al., 2022). MTT analysis showed that dopamine modification improved the cell survival and cell adhesion.

In order not to consume amino groups with antibacterial and hemostatic effects, carboxymethyl CS(CMCS) was synthesized by grafting monochloroacetic acid on the hydroxyl group of CS (Bi et al., 2022; Huang et al., 2022; Rao et al., 2022; Rao et al., 2023; Suneetha et al., 2023). Guo et al. prepared a series of high-strength composite hemostatic cryogel based on poly (vinyl alcohol) (PVA), carboxymethyl CS (CMCS), and DA by a foaming reaction and cry-polymerization reaction to cope with lethal non-compressible bleeding (Huang et al., 2022). The cryogel exhibited compression stress and can withstand a weight of 1 kg without breaking. The fungal mushroom-derived carboxymethyl CS-PDA hydrogels (FCMCS-PDA) with multifunctionality (tissue adhesive, hemostasis, self-healing, and antibacterial properties) were developed for wound dressing applications by Rao and Suneetha group, respectively (Rao et al., 2022; Rao et al., 2023; Suneetha et al., 2023).

Muco-adhesion occurs in two stages, namely, the contact stage and consolidation stage. The charge interaction between CS and mucin is reversible, while catechol-mediated interactions would provide irreversible anchorage to mucin in the consolidation stage. So hydrocaffeic acid (HCA) is often used to connect to the CS chain directly as a good candidate catechol. The grafting of HCA onto CS is easy to operate. Lee et al., have conducted a lot of work in this research (Ryu et al., 2011; Kim et al., 2013; Lee et al., 2015; Shin et al., 2017; Zhang RL. et al., 2019; Park et al., 2019; Xu et al., 2019; Shin et al., 2021), and they have developed robust tissue adhesive hydrogels consisting of catechol-functionalized CS which was obtained by HCA grafting onto CS via formation amide and thiol-terminated Pluronic F-127 in 2011 (Ryu et al., 2011). The hydrogels with remnant catechol groups showed strong adhesiveness to soft tissues and mucous layers and also demonstrated superior hemostatic properties. CS-catechol conjugates were obtained by a carbodiimide coupling method using 3, 4-dihydroxy-hydroxycinnamic acid as a catechol donor subsequently (Kim et al., 2013). This one-step chemical modification of high-molecular-weight CS (approximately 100 kDa) dramatically increased the water solubility of the CS derivative to 60 mg/mL at pH 7.0. They then prepared a hemostatic hypodermic needle coated with partially crosslinked catechol-functionalized CS that undergoes a solid-to-gel phase transition *in situ* to seal the punctured tissue. In addition, 100% of hemophilia mice survived jugular injection. This adhesive coating of self-sealing hemostatic needles may help prevent bleeding-related complications in more clinical settings (Shin et al., 2017; Shin et al., 2021). Moreover, a catechol-conjugated glycol CS was proposed as an alternative hemostatic hydrogel with negligible immune responses, enabling the replacement of CS-catechol (Park et al., 2019). The addition of ethylene glycol did not significantly modify the adhesive properties and hemostatic ability of the hydrogel but dramatically reduced the immune response. Antibacterial activity is also essential for qualified wound healing. The quaternized CS was chosen to be modified by HCA due to its respectable antibacterial, blood cell adhesion, and hemostasis (Zheng et al., 2020; Li L. et al., 2023; Wang et al., 2023). The obtained catechol-functionalized CS could combine with other substances to prepare hemostatic materials such as cotton dressing, injective hydrogels, and some materials that were even stimulus-responsive. 3,4-Dihydroxyphenylacetic acid (DOPAC) is another candidate catechol; Yin et al. prepared catechol–hydroxybutyl CS (HBCS-C) by grafting hydroxybutyl groups and DOPAC to the CS backbones (Shou et al., 2020). In this procedure, a thermo-responsive CS-based hydrogel was obtained as an injectable therapy approach for tissue adhesion and hemostasis. Ren et al. also obtained catechol-functionalized CS hydrogels by grafting CS with DOPAC (Guo et al., 2015). Tannic acid (TA) contains a great number of pyrogallol and catechol units and possesses antioxidative, anticarcinogenic, antimutagenic, and antibacterial performances, and it is a cheap natural dendritic polyphenol (Quideau et al., 2011). Similar to dopamine, TA can generate poly (tannic acid) (PTA) to graft with CS backbone. Gallic acid was conjugated to CS through the chemical modification of the primary amino groups of CS (Oh et al., 2015; Ho et al., 2018; Sanandiya et al., 2019; Huang et al., 2022). These materials all had very strong tissue adhesion and gave full play to the properties of catechol to CS-based biomaterials.

## 3 General method for preparation of mussel-inspired CS-based biomaterials

Catechins or polyphenols and their derivatives react with CS or its compounds in a variety of approaches, not only through hydrogen bonding, π–π stacking, cation-π interaction, and coordination with metal oxides but also through Schiff base bonds or Michael addition reaction (Ejima et al., 2013; Rodriguez et al., 2015; Gebbie et al., 2017; Waite, 2017; Patil et al., 2018). In summary, the general method can be roughly divided into three categories, namely, chemical cross-linking, metal–ion cross-linking, and hybrid cross-linking.

### 3.1 Chemical cross-linking

**FIGURE 1:**
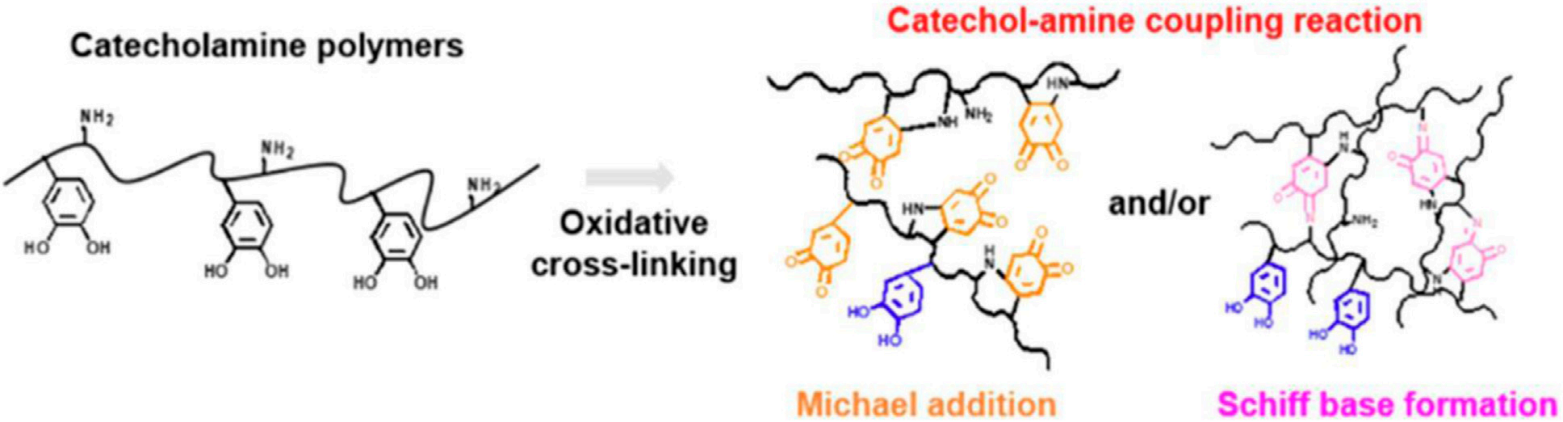
Oxidative coupling pathway of catecholamine polymers through Michael addition and/or Schiff base formation (Shin et al., 2021).

The chemical cross-linking method is the most common method for the preparation of mussel-inspired CS-based biomaterials, which can be chemically crosslinked through the following two ways, of which one is direct coupling between amine and carboxyl groups; “grafting catechins, or polyphenols and their derivatives to CS backbone” is basically linked in this way. All HCA, DOPAC, and TA use their carboxyl groups to react with amino groups of CS chains. DA can also be linked to modified CS in this way (Han et al., 2020). However, the ultimate formation of CS-based hemostatic materials is not so simple, and there are many reactions during the preparation process, which is the second way of cross-linking. The other way is through alkaline pH, NaOH, NaIO_4_, enzymes, or catalysts to promote the oxidation of catechol groups to form benzoquinone, and then, with other amine groups, catechol groups by Schiff base bonds, or Michael addition reaction to form CS-based materials base-on performance needed. “PDA or other products of self-polymerization modified CS” usually attaches PDA to a skeleton of a CS in this way. Gao’s group verified that PDA NPs could be used as a building block to cross-link with amine-rich glycol CS (GCS) through Schiff base reaction and/or Michael addition to form an injectable hydrogel (Gao et al., 2019) and that the aromatic rings on the surface of PDA NPs can adsorb a variety of drugs (ciprofloxacin etc.) via π–π stacking and/or hydrogen bonding interactions, thus giving the CS-based hemostatic materials more excellent antibacterial activity and wound healing. For mussel-inspired CS-based biomaterials, the amines of them are cross-linked to quinones by Michael addition and/or Schiff base formation has not been determined until now. To date, the consensus on the catechol-amine coupling reactions has been that they coexist in a mixed mode and are difficult to decouple from each other. Lee et al., conducted an in-depth study of the effect of temperature on the reaction between Michael addition and/or Schiff base formation (Shin et al., 2021). They found that for high-temperature oxidation (i.e., 60°C), Michael addition was a dominant oxidative coupling reaction, which weakened the CS-catechol attachment force on the needle surface. In contrast, during low-temperature oxidation (4°C), Schiff base formation was dominant, which strengthened the film attachment force on the needle surface, resulting in continued bleeding, owing to a dearth of tissue transfer after the injection, as shown in Figure 1.

### 3.2 Metal ion cross-linking

The cross-linking method of metal ions is mainly through the cross-linking of metal ions and catechol groups through the complexation (Guo et al., 2015; Yavvari and Srivastava, 2015; Fan et al., 2016; María et al., 2019). Fan et al., devised a mussel-inspired hydrogel with an easy-to-use double cross-linking mechanism by using metal ion cross-linking method, as shown in Supplementary Figure S3 (Fan et al., 2016). The first layer of cross-linking was achieved by the interaction of CS modified by catechol with Fe^3+^. Then, genipine was used to cross-link the exposed amino groups of CS to realize the internal cross-linking of the second layer. This double-cross-linked hydrogel has good biocompatibility and tissue adhesion. The similar study was carried out by dissolving HCA modified CS in ethanol/water 1:1 solution to coordinate with Fe^3+^ to obtain an interpenetrated polymer network (IPN) (María et al., 2019). Metal–ligand complexation of the catechol groups of catechol-functionalized CS present in the network with the ferric cation 20 mM was reached. In another study, CS modified with high substitution of catechol (70% substitution) explored the effect of pH on the reaction of Fe^3+^ with catechol. The hydrogels induced by Fe^3+^ were essentially a double cross-linked system consisting of covalent cross-linking and coordination cross-linking under acidic conditions, and the preparation of hydrogels induced by Fe^3+^ was a dynamic reversible process (Guo et al., 2015).

### 3.3 Hybrid cross-linking

Hybrid cross-linking was developed to combine the advantages of chemical cross-linking with physical cross-linking. Chemical cross-linked hydrogels have good mechanical strength and stability, but their gumming speed is relatively slow, and they are not sensitive to stimulation (Xu et al., 2015), which can be significantly improved by hybrid cross-linking. A kind of bio-adhesive hydrocaffeic acid-modified CS colloidal particles (HCA-CS/TPP CPs) containing synthetic catecholamine groups was prepared via application of the pickering emulsions stabilized (Zhang RL. et al., 2019). Cucurbit was employed as a non-covalent linker to facilitate interactions between catechol-functionalized CS (CAT-CS) and superparamagnetic γ-Fe_2_O_3_ NPs to enhance interactions between the two species (Qiao et al., 2019). Li H. et al. (2022) reported a novel CS-poly (ethylene glycol)-hydrocaffeic acid (CS-PEG-HA) hybrid hydrogel with the double-network cross-linked. The first network was obtained by the oxidation reaction of CS-HA using NaIO_4_; then, the secondary cross-linking occurred between dibenzocyclooctyne (DBCO)-functionalized CS-HA and four-arm PEG tetrazide. This dual-component hydrogel integrates the adhesive nature of the catechol group, the good mechanical properties of PEG, and the biocompatibility of the CS material. It is hypothesized that the incorporation of catechol and PEG groups might enable the CS hybrid hydrogel to overcome the limitations to traditional commercial wound dressings and rapid hemostasis. An NIR light-activated multifunctional hydrogel was developed based on the dynamic reversible borate and hydrogen bonds cross-linking between quaternization CS derivatives and alternatively containing phenylboronic acid and polydopamine (Peng et al., 2024).

## 4 Conclusion and outlook

**FIGURE 2:**
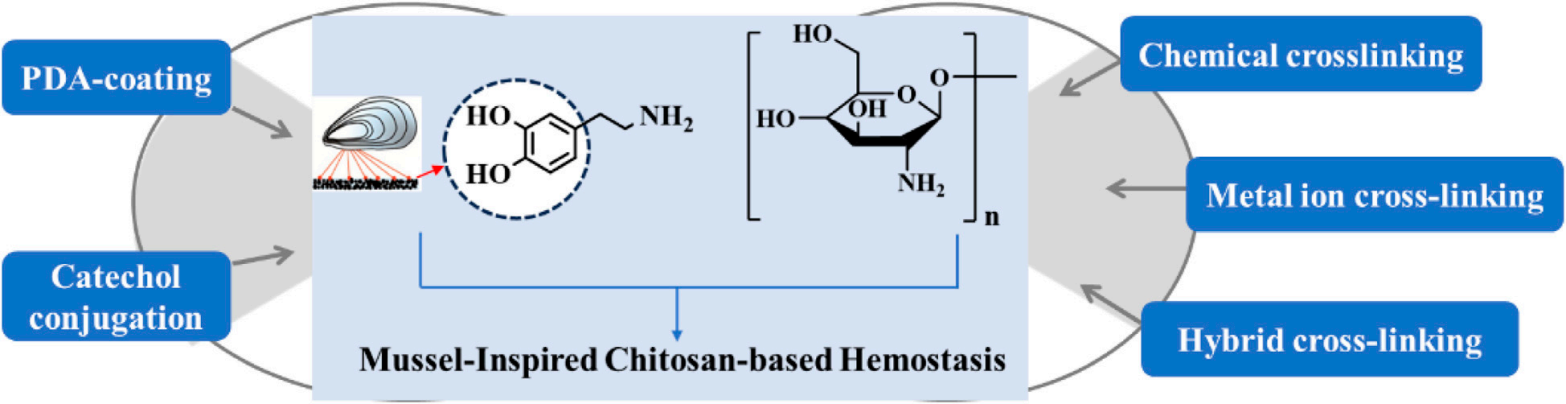
Summary and illustration of current preparation strategies of mussel-inspired CS-based biomaterials for hemostasis.

Mussel-inspired CS-based biomaterials have several advantages, such as biocompatibility, biodegradability, antibacterial activity, applicability in various formulations with hydrogels, sponges, and bandages, as well as various chemical modifications with hydrophilic and hydrophobic groups (Shokrani et al., 2022; Li WC. et al., 2023; Yang et al., 2023; Han et al., 2024). The preparation of CS-based hemostatic materials has been the focus of scientific workers is summarized in Figure 2. However, the physicochemical and biological properties of chitin cannot be precisely controlled because they depend on its biological origin, molecular weight, and degree of acetylation. This has also led to a significant gap between research on mussel-inspired CS hemostatic materials and clinically approved products, and in order to address this issue, scientists should also fully consider the adhesion mechanism and clinical limitations to the materials. In addition, modern medicine requires new smart CS-based biomaterials such as external stimuli-responsive materials, so the mussel-inspired strategy will continuously attract increasing attention in CS modification for biomedicine.

## References

[B1] Bi Z. J. Teng H. F. Li Q. J. Zhang S. K. (2022). Enhanced carboxymethylcellulose sponge for hemostasis and woundrepair. Front. Mater. 9, 944274. 10.3389/fmats.2022.944274

[B2] Deng P. P. Liang X. Chen F. X. Chen Y. Zhou J. P. (2022). Novel multifunctional dual-dynamic-bonds crosslinked hydrogels for multi-strategy therapy of MRSA-infected wounds. Appl. Mater. Today 26, 101362. 10.1016/j.apmt.2022.101362

[B3] Dreyer D. R. Miller D. J. Freeman B. D. Paul D. R. Bielawski C. W. (2013). Perspectives on poly(dopamine). Chem. Sci. 4, 3796–3802. 10.1039/c3sc51501j

[B4] Ejima H. R. J. Liang K. Best J. Koeverden M. Such G. Cui J. (2013). One-step assembly of coordination complexes for versatile film and particle engineering. Science 341, 154–157. 10.1126/science.1237265 23846899

[B5] Fan C. Fu J. Zhu W. Wang D. A. (2016). A mussel-inspired double-crosslinked tissue adhesive intended for internal medical use. Acta Biomater. 33, 51–63. 10.1016/j.actbio.2016.02.003 26850148

[B6] Fang We Yang L. Chen Y. Hu Q. L. (2023). Bioinspired multifunctional injectable hydrogel for hemostasis and infected wound management. Acta Biomater. 161, 50–66. 10.1016/j.actbio.2023.01.021 36640951

[B7] Faure E. Falentin-Daudre C. Jerome C. Lyskawa J. Fournier D. Woisel P. (2013). Catechols as versatile platforms in polymer chemistry. Prog. Polym. Sci. 38, 236–270. 10.1016/j.progpolymsci.2012.06.004

[B8] Gao G. Jiang Y. W. Jia H. R. Wu F. G. (2019). Near-infrared light-controllable on-demand antibiotics release using thermo-sensitive hydrogel-based drug reservoir for combating bacterial infection. Biomaterials 188, 83–95. 10.1016/j.biomaterials.2018.09.045 30339942

[B9] Gebbie M. A. Wei W. Schrader A. M. Cristiani T. R. Dobbs H. A. Idso M. (2017). Tuning underwater adhesion with cation-π interactions. Nat. Chem. 9 (5), 473–479. 10.1038/nchem.2720 28430190

[B10] Guo Z. W. Ni K. F. Wei D. Z. Ren Y. H. (2015). Fe^3+^-induced oxidation and coordination cross-linking in catechol-chitosan hydrogels under acidic pH conditions. RSC Adv. 5, 37377–37384. 10.1039/c5ra03851k

[B11] Han G. Y. Kwack H. W. Kim Y. H. Je Y. H. Kim H. J. Cho C. S. (2024). Progress of polysaccharide-based tissue adhesives. Carbohydr. Polym. 327, 121634. 10.1016/j.carbpol.2023.121634 38171653

[B12] Han W. Zhou B. Yang K. Wang R. Li S. Xu H. (2020). Biofilm-inspired adhesive and antibacterial hydrogel with tough tissue integration performance for sealing hemostasis and wound healing. Bioact. Mater. 5, 768–778. 10.1016/j.bioactmat.2020.05.008 32637741 PMC7317234

[B13] He Q. Gong K. Ao Q. Ma T. Yan Y. Gong Y. (2013). Positive charge of chitosan retards blood coagulation on chitosan films. J. Biomater. Appl. 27 (8), 1032–1045. 10.1177/0885328211432487 22207609

[B14] He X. Y. Sun A. Li T. Qian Y. J. Qian H. Ling Y. F. (2020). Mussel-inspired antimicrobial gelatin/chitosan tissue adhesive rapidly activated *in situ* by H(2)O(2)/ascorbic acid for infected wound closure. Carbohydr. Polym. 247, 116692. 10.1016/j.carbpol.2020.116692 32829820

[B15] Hickman D. A. Pawlowski C. L. Sekhon U. D. S. Marks J. Gupta A. S. (2018). Biomaterials and advanced technologies for hemostatic management of bleeding. Adv. Mater. 30, 1700859. 10.1002/adma.201700859 PMC5831165 29164804

[B16] Ho C. J. Seung L. J. Jisoo S. Je J. E. Soohwan A. Sun C. Y. (2018). Ascidian-inspired fast-forming hydrogel system for versatile biomedical applications: pyrogallol chemistry for dual modes of crosslinking mechanism. Adv. Funct. Mater. 28 (6), 1705244. 10.1002/adfm.201705244

[B17] Huang L. Liu M. Y. Huang H. Y. Wen Y. Q. Zhang X. Y. Wei Y. (2018). Recent advances and progress on melanin-like materials and their biomedical applications. Biomacromolecules 19, 1858–1868. 10.1021/acs.biomac.8b00437 29791151

[B18] Huang Y. ZhaoGuo X. B. L. Chen J. Liang Y. Li Z. (2022). High-strength anti-bacterial composite cryogel for lethal noncompressible hemorrhage hemostasis: synergistic physical hemostasis and chemical hemostasis. Chem. Eng. J. 427, 131977. 10.1016/j.cej.2021.131977

[B19] Kim K. Ryu J. H. Lee D. Y. Lee H. (2013). Bio-inspired catechol conjugation converts water-insoluble chitosan into a highly water-soluble, adhesive chitosan derivative for hydrogels and LbL assembly. Biomaterials Sci. 1, 783–790. 10.1039/c3bm00004d 32481831

[B20] Lee D. W. Lim C. Israelachvili J. N. Hwang D. S. (2013). Strong adhesion and cohesion of chitosan in aqueous solutions. Langmuir 29, 14222–14229. 10.1021/la403124u 24138057 PMC3888206

[B21] Lee H. Dellatore S. M. Miller W. M. Messersmith P. B. (2007). Mussel-inspired surface chemistry for multifunctional coatings. Science 318, 426–430. 10.1126/science.1147241 17947576 PMC2601629

[B22] Lee J. M. Ryu J. H. Kim E. A. Lee H. Im G. (2015). Adhesive barrier/directional controlled release for cartilage repair by endogenous progenitor cell recruitment. Biomaterials 39, 173–181. 10.1016/j.biomaterials.2014.11.006 25468369

[B23] Li F. F. Liu X. Chen F. Fu Q. (2021). Mussel-inspired chemistry: a promising strategy for natural polysaccharides in biomedical applications. Prog. Polym. Sci. 123, 101472. 10.1016/j.progpolymsci.2021.101472

[B24] Li H. Zhou X. Luo L. Ding Q. Tang S. (2022b). Bio-orthogonally crosslinked catechol-chitosan hydrogel for effective hemostasis and wound healing. Carbohydr. Polym. 281, 119039. 10.1016/j.carbpol.2021.119039 35074104

[B25] Li J. Sun X. J. Zhang K. C. Yang G. M. Mu Y. Z. Chen X. G. (2020). Chitosan/diatom-biosilica aerogel with controlled porous structure for rapid hemostasis. Adv. Healthc. Mater. 9, 2000951. 10.1002/adhm.202000951 33006258

[B26] Li L. ChenMao D. F. H. L. Ruan Z. W. Cai X. J. Jin T. (2023a). Gelatin and catechol-modified quaternary chitosan cotton dressings with rapid hemostasis and high-efficiency antimicrobial capacity to manage severe bleeding wounds. Mater. and Des. 229, 111927. 10.1016/j.matdes.2023.111927

[B27] Li W. C. Su Z. N. Hu Y. R. Meng L. H. Zhu F. Xie B. (2023b). Mussel-inspired methacrylated gelatin-dopamine/quaternized chitosan/glycerin sponges with self-adhesion, antibacterial activity and hemostatic ability for wound dressings. Int. J. Biol. Macromol. 241, 124102. 10.1016/j.ijbiomac.2023.124102 36958445

[B28] Li Y. Fu R. Z. Duan Z. G. Zhu C. H. Fan D. D. (2022a). Mussel-inspired adhesive bilayer hydrogels for bacteria-infected wound healing via NIR-enhanced nanozyme therapy. Colloids Surfaces B Biointerfaces 210, 112230. 10.1016/j.colsurfb.2021.112230 34871820

[B29] Liu K. Dong X. Z. Wang Y. Wu X. P. Dai H. L. (2022). Dopamine-modified chitosan hydrogel for spinal cord injury. Carbohydr. Polym. 298, 120047. 10.1016/j.carbpol.2022.120047 36241313

[B30] Liu Y. L. Ai K. L. Lu L. H. (2014). Polydopamine and its derivative materials: synthesis and promising applications in energy, environmental, and biomedical fields. Chem. Rev. 114, 5057–5115. 10.1021/cr400407a 24517847

[B31] María P. B. Lorena B. G. Stephanie F. Joachim K. Blanca V. L. Julio S. R. (2019). Bioadhesive functional hydrogels: controlled release of catechol species with antioxidant and anti-inflammatory behavior. Mater. Sci. Eng. C 105, 110040. 10.1016/j.msec.2019.110040 31546368

[B32] Nainggolan I. Nasution T. I. Putri S. R. E. Azdena D. Balyan M. Agusnar H. (2018). Study on chitosan film properties as a green dielectric. IOP Conf. Ser. Mater. Sci. Eng. 309, 012081. 10.1088/1757-899x/309/1/012081

[B33] Oh D. X. Kim S. Lee D. Hwang D. S. (2015). Tunicate-mimetic nanofibrous hydrogel adhesive with improved wet adhesion. Acta Biomater. 20, 104–112. 10.1016/j.actbio.2015.03.031 25841348

[B34] Ong S. Y. Wu J. Moochhal S. M. Tan M. H. Lu J. (2008). Development of a chitosan-based wound dressing with improved hemostatic and antimicrobial properties. Biomaterials 29, 4323–4332. 10.1016/j.biomaterials.2008.07.034 18708251

[B35] Park E. Lee J. Huh K. M. Lee S. H. Lee H. (2019). Toxicity-attenuated glycol chitosan adhesive inspired by mussel adhesion mechanisms. Adv. Healthc. Mater 8, 1900275. 10.1002/adhm.201900275 31091015

[B36] Patil N. Jerome C. Detrembleur C. (2018). Recent advances in the synthesis of catechol-derived (bio)polymers for applications in energy storage and environment. Prog. Polym. Sci. 82, 34–91. 10.1016/j.progpolymsci.2018.04.002

[B37] Peng W. L. Li L. X. Zhang Y. Su H. Jiang X. Liu H. (2024). Photothermal synergistic nitric oxide controlled release injectable self-healing adhesive hydrogel for biofilm eradication and wound healing. J. Mater. Chem. B 12, 158–175. 10.1039/d3tb02040a 38054356

[B38] Qiao H. S. Jia J. Chen W. Di B. Scherman O. A. Hu C. (2019). Magnetic regulation of thermo-chemotherapy from a cucurbit[7]uril-crosslinked hybrid hydrogel. Adv. Healthc. Mater. 8 (2), 1801458. 10.1002/adhm.201801458 30548830

[B39] Qiao L. P. Liang Y. P. Chen J. Y. Huang Y. Alsareii S. A. Alamri A. M. (2023). Antibacterial conductive self-healing hydrogel wound dressing with dual dynamic bonds promotes infected wound healing. Bioact. Mater. 30, 129–141. 10.1016/j.bioactmat.2023.07.015 37554541 PMC10404845

[B40] Qu J. Zhao X. Liang Y. P. Zhang T. L. Ma P. X. Guo B. L. (2018). Antibacterial adhesive injectable hydrogels with rapid self-healing, extensibility and compressibility as wound dressing for joints skin wound healing. Biomaterials 183, 185–199. 10.1016/j.biomaterials.2018.08.044 30172244

[B41] Quideau S. Deffieux D. Douat C. C. Pouysegu L. (2011). Plant polyphenols: chemical properties, biological activities, and synthesis. Angew. Chem. Int. Ed. 50, 586–621. 10.1002/anie.201000044 21226137

[B42] Rao K. M. Badri N. K. Han S. S. Park P. H. Choi I. (2022). Tissue adhesive, self-healing, biocompatible, hemostasis, and antibacterial properties of fungal-derived carboxymethyl chitosan-polydopamine hydrogels. Pharmaceutics 14 (5), 1028. 10.3390/pharmaceutics14051028 35631614 PMC9145872

[B43] Rao K. M. Uthappa U. T. Kim H. J. Han S. S. (2023). Tissue adhesive, biocompatible, antioxidant, and antibacterial hydrogels based on tannic acid and fungal-derived carboxymethyl chitosan for wound-dressing applications. Gels 9 (5), 354. 10.3390/gels9050354 37232946 PMC10217669

[B44] Rao S. B. Sharma C. P. (1997). Use of chitosan as a biomaterial: studies on its safety and hemostatic potential. J. Biomed. Mater. Res. 34 (1), 21–28. 10.1002/(sici)1097-4636(199701)34:1<21::aid-jbm4>3.0.co;2-p 8978649

[B45] Rodriguez N. R. M. Das S. Kaufman Y. Israelachvili J. N. Waite J. H. (2015). Interfacial pH during mussel adhesive plaque formation. Biofouling 31 (2), 221–227. 10.1080/08927014.2015.1026337 25875963 PMC4420479

[B46] Ryu J. H. Lee Y. Kong W. H. Kim T. G. Park T. G. Lee H. (2011). Catechol-functionalized chitosan/pluronic hydrogels for tissue adhesives and hemostatic materials. Biomacromolecules 12, 2653–2659. 10.1021/bm200464x 21599012

[B47] Sanandiya N. D. Lee S. Y. Rho S. Lee H. Kim I. S. Hwang D. S. (2019). Tunichrome-inspired pyrogallol functionalized chitosan for tissue adhesion and hemostasis. Carbohydr. Polym. 208, 77–85. 10.1016/j.carbpol.2018.12.017 30658834

[B48] Shin M. Choi J. H. Kim K. Kim S. Lee H. (2021). Hemostatic needles: controlling hemostasis time by a catecholamine oxidative pathway. ACS Appl. Mater. Interfaces 13, 10741–10747. 10.1021/acsami.0c22223 33620191

[B49] Shin M. Park S. G. Oh B. C. Kim K. Jo S. Lee M. S. (2017). Complete prevention of blood loss with self-sealing haemostatic needles. Nat. Mater. 16, 147–152. 10.1038/nmat4758 27698353

[B50] Shokrani H. Shokrani A. Seidi F. Kucinska-Lipka J. Saeb M. Fatahi Y. (2022). Biomedical engineering of polysaccharide-based tissue adhesives: recent advances and future direction. Carbohydr. Polym. 295, 119787. 10.1016/j.carbpol.2022.119787 35989028

[B51] Shou Y. F. Zhang J. H. Yan S. F. Xia P. F. Xu P. L. Li G. F. (2020). Thermoresponsive chitosan/DOPA-based hydrogel as an injectable therapy approach for tissue-adhesion and hemostasis. ACS Biomater. Sci. Eng. 6, 3619–3629. 10.1021/acsbiomaterials.0c00545 33463168

[B52] Simpson A. Shukla A. Brown A. C. (2022). Biomaterials for hemostasis. Biomaterials Hemostasis 24, 111–135. 10.1146/annurev-bioeng-012521-101942 PMC9177659 35231178

[B53] Singh G. Nayal A. Malhotra S. Koul V. (2020). Dual functionalized chitosan based composite hydrogel for haemostatic efficacy and adhesive property. Carbohydr. Polym. 247, 116757. 10.1016/j.carbpol.2020.116757 32829870

[B54] Singha G. Nayal A. Sahil M. Koul V. (2020). Dual functionalized chitosan based composite hydrogel for haemostatic efficacy and adhesive property. Carbohydr. Polym. 247, 116757. 10.1016/j.carbpol.2020.116757 32829870

[B55] Sun C. Y. Zeng X. L. Zheng S. H. Wang Y. L. Li Z. Y. Zhang H. M. (2022a). Bio-adhesive catechol-modified chitosan wound healing hydrogel dressings through glow discharge plasma technique. Chem. Eng. J. 427, 130843. 10.1016/j.cej.2021.130843

[B56] Sun W. Mu C. J. Shi H. C. Yan Q. Y. Luan S. F. (2022b). Mussel-inspired polysaccharide-based sponges for hemostasis and bacteria infected wound healing. Carbohydr. Polym. 295, 119868. 10.1016/j.carbpol.2022.119868 35989011

[B57] Suneetha M. Zo S. Choi S. M. Han S. S. (2023). Antibacterial, biocompatible, hemostatic, and tissue adhesive hydrogels based on fungal-derived carboxymethyl chitosan-reduced graphene oxide-polydopamine for wound healing applications. Int. J. Biol. Macromol. 241, 124641. 10.1016/j.ijbiomac.2023.124641 37119909

[B58] Tao B. L. Lin C. C. Yuan Z. Chen M. W. Li K. Hu J. W. (2021). Near infrared light-triggered on-demand Cur release from Gel-PDA @Cur composite hydrogel for antibacterial wound healing. Chem. Eng. J. 403, 126182. 10.1016/j.cej.2020.126182

[B59] Waite H. J. (2017). Mussel adhesion-essential footwork. J. Exp. Biol. 220 (4), 517–530. 10.1242/jeb.134056 28202646 PMC5312731

[B60] Wang L. L. Zhao Z. Q. Dong J. H. Li D. W. Liu Q. S. Deng B. Y. (2023). Mussel-inspired multifunctional hydrogels with adhesive, self-healing, antioxidative, and antibacterial activity for wound healing. ACS Appl. Mater. Interfaces 15, 16515–16525. 10.1021/acsami.3c01065 36951622

[B61] Wang Y. Y. Fu Y. M. Feng C. Chen X. G. Zhang X. Zhang K. (2018). Multifunctional chitosan/dopamine/diatom-biosilica composite beads for rapid blood coagulation. Carbohydr. Polym. 200, 6–14. 10.1016/j.carbpol.2018.07.065 30177204

[B62] Xu J. Strandman S. Zhu J. X. X. Barralet J. Cerruti M. (2015). Genipin-crosslinked catechol-chitosan mucoadhesive hydrogels for buccal drug delivery. Biomaterials 37, 395–404. 10.1016/j.biomaterials.2014.10.024 25453967

[B63] Xu R. N. Ma S. H. Wu Y. Lee H. Zhou F. Liu W. M. (2019). Adaptive control in lubrication, adhesion, and hemostasis by Chitosan-Catechol-pNIPAM. Biomaterials Sci. 7, 3599–3608. 10.1039/c9bm00697d 31339146

[B64] Yang E. Hou W. Liu K. Wei W. Y. Kang H. F. Dai H. L. (2022). A multifunctional chitosan hydrogel dressing for liver hemostasis and infected wound healing. Carbohydr. Polym. 291, 119631. 10.1016/j.carbpol.2022.119631 35698421

[B65] Yang Y. Y. Ma Y. Wang J. F. You L. R. Zhang R. T. Meng Q. Y. (2023). Chitosan-based mussel-inspired hydrogel for rapid self-healing and high adhesion of tissue adhesion and wound dressings. Carbohydr. Polym. 316, 121083. 10.1016/j.carbpol.2023.121083 37321753

[B66] Yavvari P. S. Srivastava A. (2015). Robust, self-healing hydrogels synthesised from catechol rich polymers. J. Mater. Chem. B 3, 899–910. 10.1039/c4tb01307g 32262181

[B67] Yin G. Wang J. Wang X. Zhan Y. Tang X. Wu Q. (2022). Multifunctional all-in-one adhesive hydrogel for the treatment of perianal infectious wounds. Front. Bioeng. Biotechnol. 10, 989180. 10.3389/fbioe.2022.989180 36246359 PMC9561363

[B68] Zhang R. L. Hao H. Zhang C. G. Yang R. C. Sun M. Y. Wong C. P. (2019a). Bioadhesive hydrocaffeic acid modified chitosan colloidal particles using as particulate emulsifiers. J. Dispersion Sci. Technol. 40 (11), 1559–1566. 10.1080/01932691.2018.1484755

[B69] Zhang X. Sun G. H. Tian M. P. Wang Y. N. Cheng X. J. Feng C. (2019b). Mussel-inspired antibacterial polydopamine/chitosan/temperature-responsive hydrogels for rapid hemostasis. Int. J. Biol. Macromol. 138, 321–333. 10.1016/j.ijbiomac.2019.07.052 31295499

[B70] Zhao X. L. Huang Y. F. Tian X. Luo J. N. Wang H. X. Wang J. F. (2022). Polysaccharide-based adhesive antibacterial and self-healing hydrogel for sealing hemostasis. Biomacromolecules 23, 5106–5115. 10.1021/acs.biomac.2c00943 36395528

[B71] Zheng Z. Q. Bian S. Q. Li Z. Q. Zhang Z. Liu Y. Zhai X. (2020). Catechol modified quaternized chitosan enhanced wet adhesive and antibacterial properties of injectable thermo-sensitive hydrogel for wound healing. Carbohydr. Polym. 249, 116826. 10.1016/j.carbpol.2020.116826 32933673

