# Supplementary material for: Preparation strategies of mussel-inspired chitosan-based biomaterials for hemostasis
Source: Front Pharmacol. 2024 Aug 15;15:1439036. doi: 10.3389/fphar.2024.1439036 (PMC11363193; doi:10.3389/fphar.2024.1439036)
Supplement: Supplementary file 1 [file DataSheet1.pdf]

Preparation Strategies of Mussel-Inspired Chitosan-based Biomaterials for Hemostasis

Guihua Cui* [a,1], Xiaoyu Guo [b,1], Li Deng* [c]

[a] Department of Chemistry, Jilin Medical University, Jilin, Jilin, 132013, China.

[b] Jilin Vocational College of Industry and Technology, Jilin, Jilin, 132013, China.

[c] Department of Extracorporeal Life Support, The People's Hospital of Gaozhou, Guangdong, China.

*Corresponding authors: Guihua Cui, E-mail: cuiyuhan1981_0@sohu.com, Fax/Tel: +86 432 64560187. Li Deng, dengli_198118@163.com.

[1] The two authors contribute equally to this work.

2.1 PDA or other products of self-polymerization modified CS

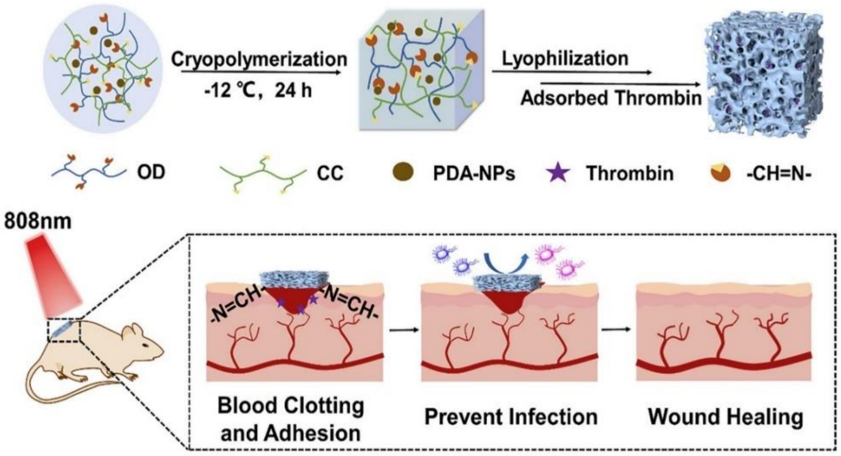

Fig.S1. CS-based sponges and their application as antibacterial and wound dressing materials[23].

2.2 Grafting catechins or polyphenols and their derivatives to CS backbone

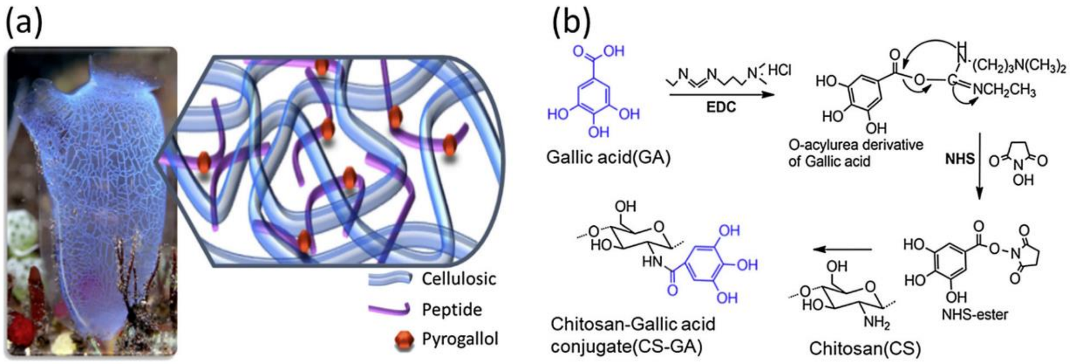

Fig.S2. Synthesis of tunichrome-inspired chitosan hydrogel. (a) Schematic illustration of the structure of tunic found in tunicates, (b) synthesis mechanism of tunichrome-inspired chitosan hydrogel by conjugation of gallic acid (CS-GA)-pyrogallol moieties by EDC coupling amidation reaction[15]

3.2 Metal ion cross-linking

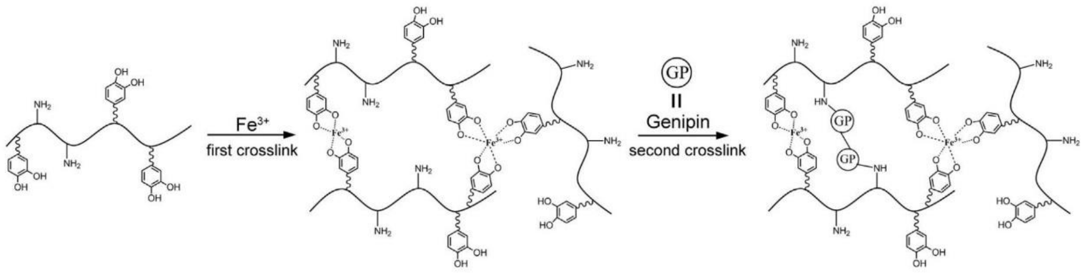

Fig.S3 Diagram of hydrogel preparation of double cross-linking mechanism[60]
